# Rising trends in dementia and psychoactive drug-related mortality in the United States, 2000–2023. A retrospective analysis using CDC WONDER

**DOI:** 10.1186/s12883-026-05117-8

**Published:** 2026-06-29

**Authors:** Unsa Arif, Abdul Moeez Awais, Eraj Nadeem, Vishan Das, Muhammad Wassam, Fatima Sohail, Shaheer Bin Shafiq, Falak Naz, Sanhia Maheshwari, Muhammad Touseef, Modibo Yattassaye

**Affiliations:** 1 Continental Medical College, Lahore, Punjab Pakistan; 2 Islamic International Medical College, Riphah International University, Rawalpindi, Pakistan; 3 Liaquat National Hospital and Medical College, Karachi, Sindh Pakistan; 4 Liaquat University of Medical & Health Sciences, Jamshoro, Sindh Pakistan; 5 Al Tibri Medical College, Isra University, Karachi, Sindh Pakistan; 6 Jinnah Sindh Medical University, Karachi, Sindh Pakistan; 7 Department of Internal Medicine, Dow University of Health Sciences, Karachi, Pakistan; 8 Richmond University Medical Center, New York, Staten Island USA; 9 University College of Medicine and Dentistry Lahore, Lahore, Punjab Pakistan; 10 Faculty of Medicine and Pharmaceutical Science, University of Douala, Douala, Cameroon

**Keywords:** Dementia, Substance use disorders, Psychoactive drugs, Mortality trends, CDC WONDER

## Abstract

**Background:**

Dementia, termed major neurocognitive disorder in the DSM-5-TR, and substance use disorders (SUD) are major and increasingly overlapping public health concerns in the United States. However, population-level trends examining their combined contribution to mortality remain limited. This study evaluates national mortality trends involving both conditions.

**Methods:**

We conducted a retrospective analysis of the CDC WONDER Multiple Cause of Death database (2000–2023). Deaths listing both dementia and SUD as underlying or contributing causes were identified using ICD-10 codes. Age-adjusted mortality rates (AAMRs) per 100,000 were calculated using the 2000 U.S. standard population. Trends were analyzed using Joinpoint regression, reporting annual percent change (APC) and average annual percent change (AAPC) with 95% confidence intervals (CIs).

**Results:**

A total of 283,206 deaths were identified. The overall AAMR increased significantly from 0.58 in 2000 to 7.63 in 2023 (AAPC: 13.29%; 95% CI: 10.42–16.23; p<0.001).

Sex-stratified analysis showed higher mortality in males (AAMR: 0.87→9.61) than females (0.41→6.23), although females demonstrated a greater relative increase (AAPC: 14.07% vs 12.55%; both *p*<0.001).

By race, non-Hispanic (NH) White individuals had the highest absolute rates (AAMR: 0.60→8.57), while NH Black individuals exhibited the highest long-term burden (AAPC: 12.69%; *p*<0.001). Hispanic individuals showed lower rates but consistent increases (AAMR: 0.22→3.33; *p*<0.001).

Age-stratified analysis demonstrated increasing age-adjusted mortality rates among individuals aged 45–64 years (0.14→0.61; *p*<0.001). Among older adults (≥65 years), mortality rates initially increased substantially (2.79→41.94) through 2020 (*p*<0.001), then declined sharply, reaching 0.61 in 2023 (*p*<0.001).

Regionally, the Midwest had the highest mortality (AAMR: 0.58→10.8; AAPC: 14.38%; *p*<0.001), while the Northeast had the lowest rates (0.29→4.95; *p*<0.001). All regions showed significant upward trends (*p*<0.001).

Urbanization analysis revealed increasing mortality in metropolitan (0.56→3.5) and non-metropolitan areas (0.71→3.8), with stronger growth in non-metropolitan regions (AAPC: 16.72% vs 15.82%; *p*<0.001).

**Conclusion:**

Mortality involving both dementia and substance use disorders has increased substantially in the United States over the past two decades, with significant disparities across sex, race, geography, and urbanization. These findings highlight the growing burdens of neurodegenerative disease and substance use, emphasizing the need for integrated public health strategies targeting both conditions.

**Supplementary Information:**

The online version contains supplementary material available at https://doi.org/10.1186/s12883-026-05117-8.

## Introduction

Dementia is defined as a syndrome characterized by gradual deterioration of nerve cells leading to a decline in cognitive function. Dementia has multiple subtypes, including Parkinson’s disease dementia, Lewy body dementia, vascular dementia, mixed dementia and Alzheimer’s disease [1]. Among these, Alzheimer’s disease alone accounts for almost half of the cases [2]. Despite growing research, dementia remains the 7th most prevalent cause of mortality worldwide, according to the WHO. About 7 million residents of the U.S are reported to be living with Alzheimer’s dementia. As of a special report in 2025, this number is estimated to increase by more than 90% by 2060 [3].

Dementia can be caused by non-modifiable factors like age, gender, race, and genetics. Prevalence increases with age; a higher incidence is observed in Older Black individuals compared to older white individuals, as well as in females compared to males [4]. Notably, a substantial portion of cases is attributable to modifiable risk factors, most prominently poor lifestyle, highlighting the importance of prevention strategies [5]. Consequently, substance use disorders (SUD) are one of the most critical threats to the lifestyles of American Society, with 48.4 million residents of the U.S aged 12 and above meeting the diagnostic criteria [6]. The `Center for Drug Abuse Statistics (NCDAS) reports rising levels of Alcohol, Cannabis, and prescription drug abuse, which have contributed to over 100,000 deaths annually [7].

Emerging evidence suggests a bidirectional relationship between substance use and Dementia. Chronic exposure to substances like alcohol, opioids, amphetamines, and tobacco has been linked to cognitive decline through mechanisms including direct neurotoxicity, cerebrovascular injury, oxidative stress, and nutritional deficiencies [8–10]. These effects may accelerate neurodegeneration and increase the risk of developing dementia. Conversely, population- based studies suggest individuals with these disorders are at a higher risk of dementia, with just chronic smoking and tobacco chewing increasing incidence by 79%, and amphetamines raising prevalence almost 4-fold [11]. Furthermore, substance use disorders are also associated with comorbid conditions like psychiatric disorders and cardiovascular diseases, which may further compound dementia risk and mortality [12, 13].

Despite growing recognition of this association, existing literature has largely focused on either dementia or substance use independently, with limited population-level analyses examining their combined contribution to mortality. Additionally, the extent to which SUD contributes to dementia- related deaths (an underlying cause, contributing factor, or comorbidity) remains insufficiently characterized.

Therefore, this study aims to examine national trends in mortality involving both substance use disorders and dementia in the United States from 2000 to 2023 using CDC WONDER data. Through this, we aim to identify demographic and regional disparities and clarify trends to develop informed, targeted interventions. This study seeks not only to address this dual burden but also to contribute to existing literature.

## Methodology

### Study setting and population

This retrospective analysis utilized data on mortality trends related to Substance Use disorders (SUD) and Dementia collected from the Centers for Disease Control and Prevention’s Wide-Ranging Online Data for Epidemiological Research (CDC WONDER) [14]. The Multiple Cause of Death Database was used to identify deaths in which both SUDs and Dementia were listed as either underlying or contributing causes. Diagnostic coding was employed using the International Classification of Diseases and Related Health Problems, 10th Revision (ICD-10) codes F01.1, F01.1, F01.2, F01.3, F01.8, F01.9, F03, G30, G31 for Dementia and F10, F11, F12, F13, F14, F15, F16, F17, F18, F19 for disorders due to psychoactive substance use [15]. These codes were in accordance with previous epidemiological studies [16, 17]. This study did not require approval from the institutional review board because it used de-identified death certificate data publicly available. Moreover, our study complied with the Strengthening the Reporting of Observational Studies in Epidemiology (STROBE) guidelines [18].

### Data extraction and variables

Mortality data from the CDC WONDER database, between 2000 and 2023, were categorized by gender, race, age group, urbanization status, census region, and state. The database included death certificate data from all 50 U.S. states and the District of Columbia. Gender was available as male or female, which was assigned at birth. Data were extracted and analysed for ages grouped into younger adults (25–44 years), middle-aged adults (45–64 years), and older adults (65 + years). However, due to unreliability, the young adults group was removed from the final analysis due to statistical unreliability, caused by low death counts for dementia-related causes in this age group, which may lead to unstable mortality rates and potential miscalculation bias. Similarly, the racial or ethnic groups were non-Hispanic (NH) White, NH Black or African, American Hispanic or Latino, NH American Indian, or Alaska Native, and NH Asian populations, but those with inconsistencies were removed from the analysis to ensure the viability of the results. Place of death was recorded as home, nursing home, hospital, hospice facility, or other. The urban-rural classification scheme was stratified into urban (including large metropolitan areas with populations more than 1 million), medium/small metropolitan areas with populations between 50,000 and 999,999, or rural with populations less than 50,000 [19]. Geographic regions were categorized according to the U.S. Census Bureau’s directions into the Northeast, Midwest, South and West [20].

### Data analysis

To analyze trends related to SUD and Dementia, Age-adjusted mortality rates (AAMR) and Crude mortality rates (CMR) per 100,000 were calculated. CMR was calculated by dividing the number of deaths by the corresponding U.S. population, while AAMR values were calculated by using the 2000 U.S. standard population [21]. Temporal trends were assessed using JointPoint Regression Analysis Software (Version 5.4.0; National Cancer Institute), which was fitted with a maximum of 4 jointpoints and was used to detect areas of significant change over time [22]. Annual percentage change (APC) and 95% confidence intervals (CIs) for AAMRs were calculated using the Monte Carlo permutation test. APC values correspond to the JointPoint-derived trends and were considered regressing or progressing by determining whether the change in the trend was statistically different from 0 using a two-tailed t-test. A value of p less than 0.05 was considered statistically significant.

The average annual percentage change (AAPC) was calculated as a weighted summary measure of APCs across all jointpoint segments, where each segment is weighted according to its duration. This provides an overall summary of trends across the entire study duration instead of just one segment. Since AAPCs are interpreted as the net trend over time, they can differ from visual trends observed in figures, particularly when trends vary across different time periods.

## Results

Between 2000 and 2023, a total of 283,206 deaths were identified in which Substance Use and Dementia were listed as either the underlying or contributing cause of death in the United States. All mortality rates are reported per 100,000 population and were age-adjusted to the 2000 U.S. standard population unless otherwise specified.

Complete information regarding the place of death was available for 283,194 decedents. The minor discrepancy of 12 deaths was attributable to CDC WONDER suppression policies for small cell counts.

### Annual trends in substance use and dementia-related AAMR

**Fig. 1:**
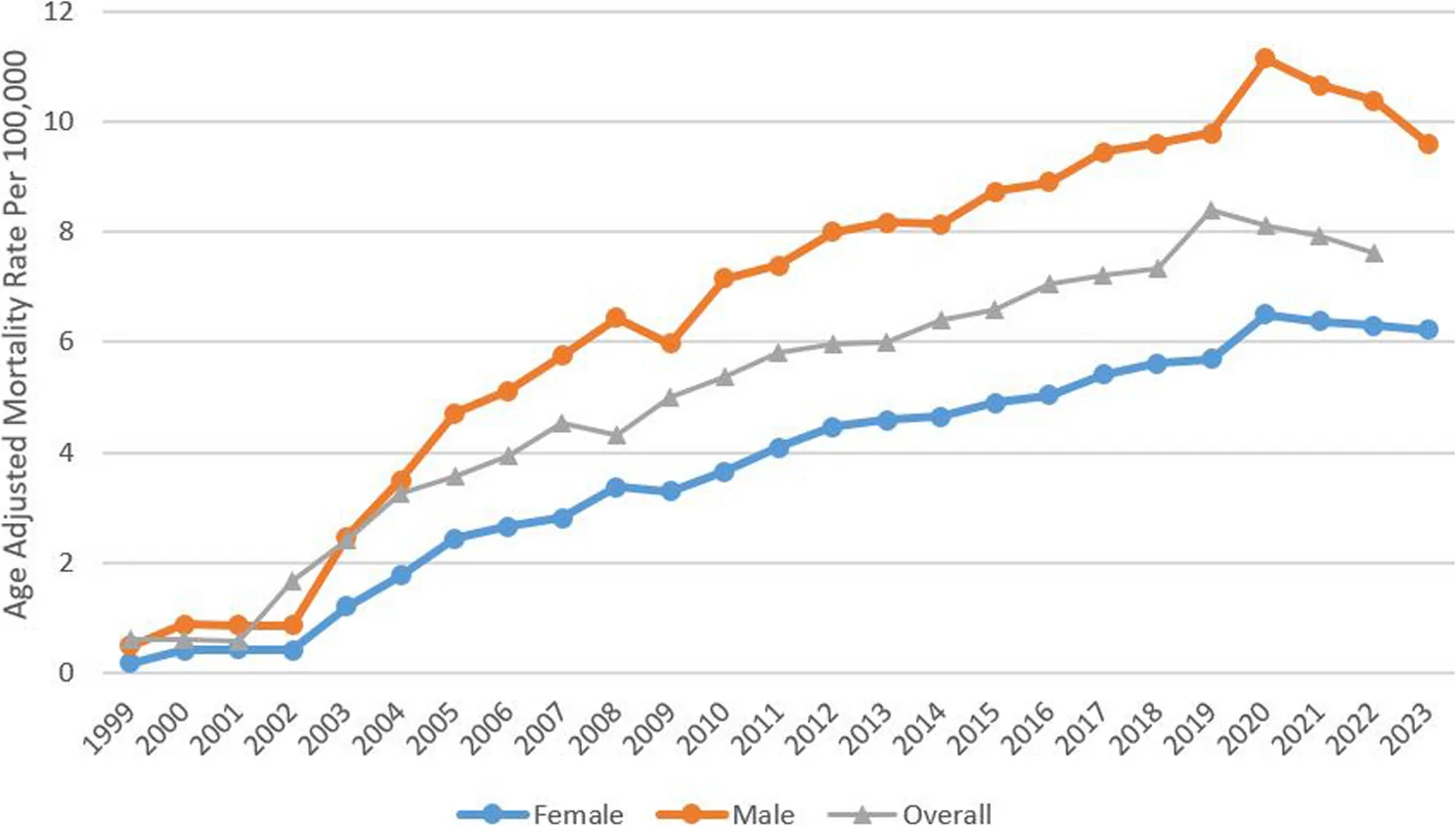
Overall, trends in psychoactive drugs and dementia-related age-adjusted mortality rates (AAMRs) per 100,000 in the United States, 2000 to 2023

As detailed in Fig. 1; Tables 1, 2 and 3, and Supplementary Table 1, from 2000 to 2023, Substance Use and Dementia-related mortality demonstrated a marked and sustained increase. The age-adjusted mortality rate (AAMR) increased from 0.58 in 2000 to 7.63 in 2023. Joinpoint regression identified a sharp rise from 2000 to 2005 (APC: 52.96%; 95% CI: 36.03 to 72.01; *p* < 0.001), followed by a continued but slower increase from 2005 to 2020 (APC: 5.62%; 95% CI: 4.71 to 6.52; *p* < 0.001). A non-significant decline was observed from 2020 to 2023 (APC: −2.48%; 95% CI: −9.27 to 4.82; *p* = 0.47). Overall, the long-term trend showed a significant upward trajectory, with an average annual percent change (AAPC) of 13.29% (95% CI: 10.42 to 16.23; *p* < 0.001).

**Table 1:** Dementia and psychoactive drug-related deaths stratified by sex among adults age in the United States, 2000 to 2023

Year	Overall	Women	Men
2000	1047	453	594
2001	1075	479	596
2002	1057	457	600
2003	3089	1397	1692
2004	4557	2112	2445
2005	6273	2923	3350
2006	7015	3265	3750
2007	7834	3511	4323
2008	9194	4222	4972
2009	8936	4219	4717
2010	10,483	4723	5760
2011	11,552	5396	6156
2012	12,867	6003	6864
2013	13,515	6275	7240
2014	13,925	6486	7439
2015	15,203	6988	8215
2016	15,958	7350	8608
2017	17,461	8025	9436
2018	18,412	8494	9918
2019	19,126	8774	10,352
2020	22,354	10,207	12,147
2021	20,424	9372	11,052
2022	21,347	9976	11,371
2023	20,502	9743	10,759
Total	283,206	130,850	152,356

**Table 2:** Dementia and psychoactive drug-related deaths stratified by race among adults age in the United States, 2000 to 2023

Year	NH Black or African American	Hispanic or Latino	NH Whites	American Indian or Alaska Native	Asian or Pacific Islander
2000	95	20	920	Suppressed	Suppressed
2001	92	30	939	11	Suppressed
2002	102	25	918	Suppressed	Suppressed
2003	218	102	2752	10	Suppressed
2004	294	114	4109	27	13
2005	438	170	5610	32	23
2006	452	227	6229	50	57
2007	516	246	6980	45	47
2008	632	242	8211	44	65
2009	622	259	7930	50	75
2010	758	301	9279	66	79
2011	758	325	10,316	68	85
2012	894	398	11,418	63	94
2013	1001	426	11,918	60	110
2014	1044	489	12,209	77	106
2015	1150	483	13,379	85	106
2016	1253	557	13,901	109	138
2017	1347	610	15,220	128	156
2018	1418	710	15,980	123	181
2019	1508	740	16,575	114	189
2020	1905	929	19,141	139	240
2021	1759	779	17,553	-	-
2022	1785	869	18,340	-	-
2023	1730	793	17,603	-	-
Total	21,771	9844	247,430	1301	1764

‘Suppressed’ indicates that the number of deaths in a specific category is between 1 and 9. In accordance with CDC WONDER sub-national data use restrictions, these values are suppressed to ensure the confidentiality of individuals and to prevent the identification of decedents

**Table 3:** Annual percent change (APC) and Average annual percent change (AAPC) are shown as age-adjusted mortality rates per 100,000 among adults age in the United States, 2000–2023

Category	Year Segment	APC	APC Lower	APC Upper	AAPC	*p*-value (APC)	*p*-value (AAPC)
Overall	2000–2005	52.9640*	36.0287	72.0077	13.2880*	0.000001	< 0.000001
	2005–2020	5.6159*	4.7149	6.5247		< 0.000001	
	2020–2023	-2.4753	-9.2669	4.8246		0.472313	
Female	2000–2005	50.3744*	34.3410	68.3215	14.0714*	0.000001	< 0.000001
	2005–2012	8.7364*	4.5794	13.0586		0.000324	
	2012–2023	3.7218*	2.6179	4.8375		0.000002	
Male	2000–2005	52.2660*	36.3674	70.0181	12.5549*	< 0.000001	< 0.000001
	2005–2020	5.0169*	4.1019	5.9400		< 0.000001	
	2020–2023	-3.8035	-10.2655	3.1238		0.254414	
45–64	2000–2023	5.5447*	4.7432	6.3523	5.5447*	< 0.000001	< 0.000001
65–85+	2000–2005	56.1811*	39.5474	74.7976	-10.0580*	< 0.000001	0.000009
	2005–2020	5.3042*	4.3327	6.2846		< 0.000001	
	2020–2023	-83.7031*	-88.3472	-77.2082		< 0.000001	
Hispanic or Latino	2000–2005	55.1157*	25.8743	91.1500	13.1899*	0.000309	< 0.000001
	2005–2023	3.7037*	2.7420	4.6744		< 0.000001	
Black or African American	2000–2005	46.3436*	31.0272	63.4505	12.6904*	0.000002	< 0.000001
	2005–2020	6.2213*	5.2013	7.2513		< 0.000001	
	2020–2023	-2.0242	-8.9419	5.4190		0.562087	
White	2000–2005	54.4150*	38.0244	72.7521	13.7722*	< 0.000001	< 0.000001
	2005–2020	5.9122*	4.9429	6.8904		< 0.000001	
	2020–2023	-2.1869	-9.2917	5.4743		0.542952	
Northeast	2000–2004	90.4800*	57.5481	130.2955	15.2617*	0.000006	< 0.000001
	2004–2007	18.9179	-4.8516	48.6254		0.117098	
	2007–2020	2.3740*	1.2686	3.4914		0.000439	
	2020–2023	-4.4160	-12.2005	4.0586		0.271417	
Midwest	2000–2008	38.1549*	27.8987	49.2336	14.3781*	< 0.000001	< 0.000001
	2008–2023	3.4182*	2.3411	4.5066		0.000002	
South	2000–2005	45.3349*	29.8047	62.7232	12.9645*	0.000003	< 0.000001
	2005–2020	6.6287*	5.6421	7.6245		< 0.000001	
	2020–2023	-0.9392	-7.9498	6.6052		0.788685	
West	2000–2005	37.9350*	24.2227	53.1609	10.8360*	0.000004	< 0.000001
	2005–2023	4.3023*	3.5939	5.0155		< 0.000001	
Metro	2000–2005	54.0137*	38.0632	71.8069	15.8234*	< 0.000001	< 0.000001
	2005–2020	5.3276*	4.4831	6.1789		< 0.000001	
Non-Metro	2000–2004	60.2419*	37.0906	87.3028	16.7166*	0.000019	< 0.000001
	2004–2012	11.0966*	7.8847	14.4042		0.000003	
	2012–2020	4.6508*	2.9327	6.3976		0.000050	

### Sex trends

**Fig. 2:**
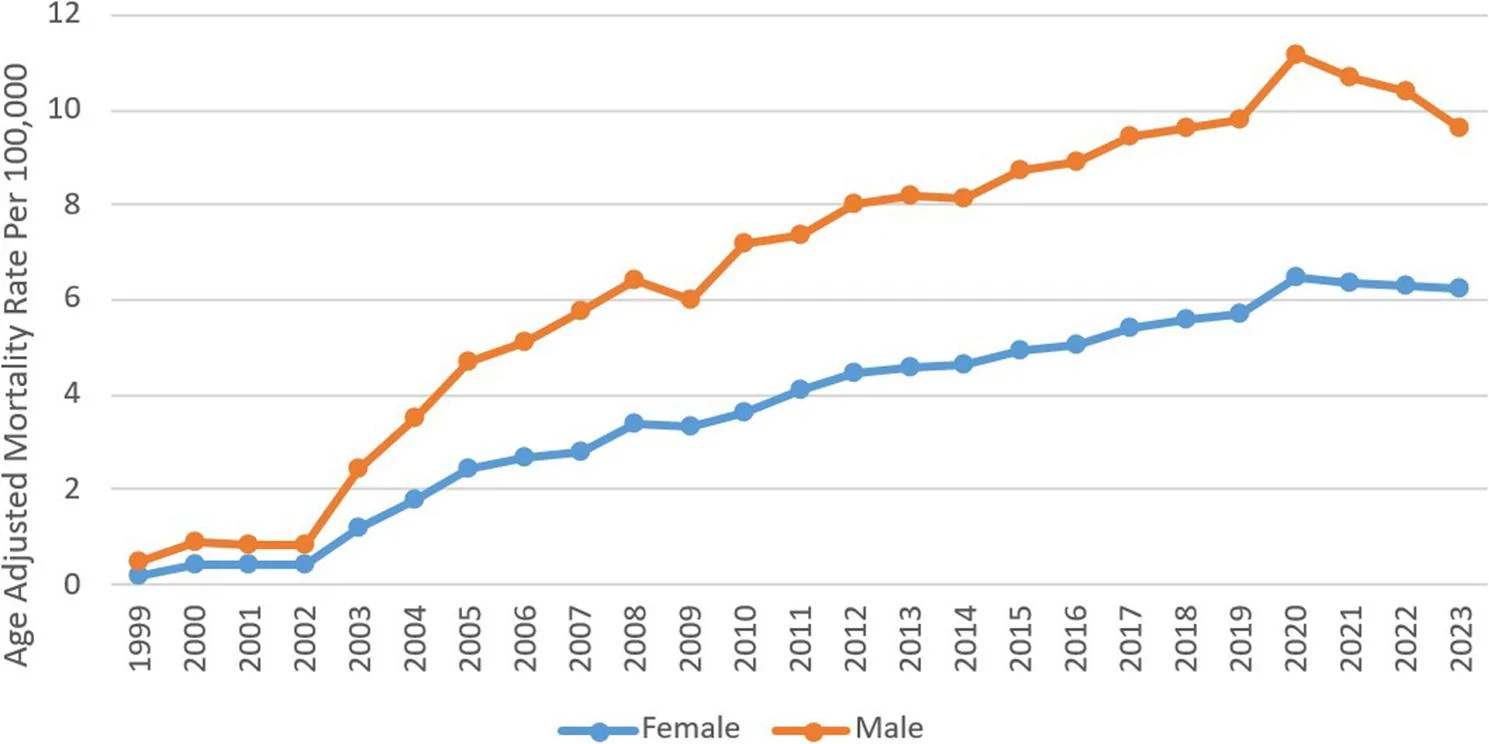
Sex-stratified psychoactive drugs and dementia-related age-adjusted mortality rates (AAMRs) per 100,000 individuals in the United States, 2000 to 2023

As illustrated in Figs. 1 and 2; Tables 1 and 2, and 3, and Supplementary Table 1, among males, the AAMR increased from 0.87 per 100,000 in 2000 to 9.61 per 100,000 in 2023. Mortality increased sharply from 2000 to 2005 (APC: 52.27%, 95% CI: 36.37 to 70.02; *p* < 0.000001), followed by a sustained rise from 2005 to 2020 (APC: 5.02%, 95% CI: 4.10 to 5.94; *p* < 0.000001). A decline was observed from 2020 to 2023 (APC: −3.80%, 95% CI: −10.27 to 3.12; *p* = 0.25). The overall trend in males remained increasing (AAPC: 12.55%, 95% CI: 9.86 to 15.32; *p* < 0.000001).

In females, AAMRs increased from 0.41 per 100,000 in 2000 to 6.23 per 100,000 in 2023. A pronounced increase occurred from 2000 to 2005 (APC: 50.37%, 95% CI: 34.34 to 68.32; *p* < 0.001), followed by continued growth from 2005 to 2012 (APC: 8.74%, 95% CI: 4.58 to 13.06; *p* < 0.001), and a sustained increase from 2012 to 2023 (APC: 3.72%, 95% CI: 2.62 to 4.84; *p* < 0.001). Consequently, females demonstrated the highest relative increase, with an AAPC of 14.07% (95% CI: 11.19 to 17.03; *p* < 0.001).

### Race trends

**Fig. 3:**
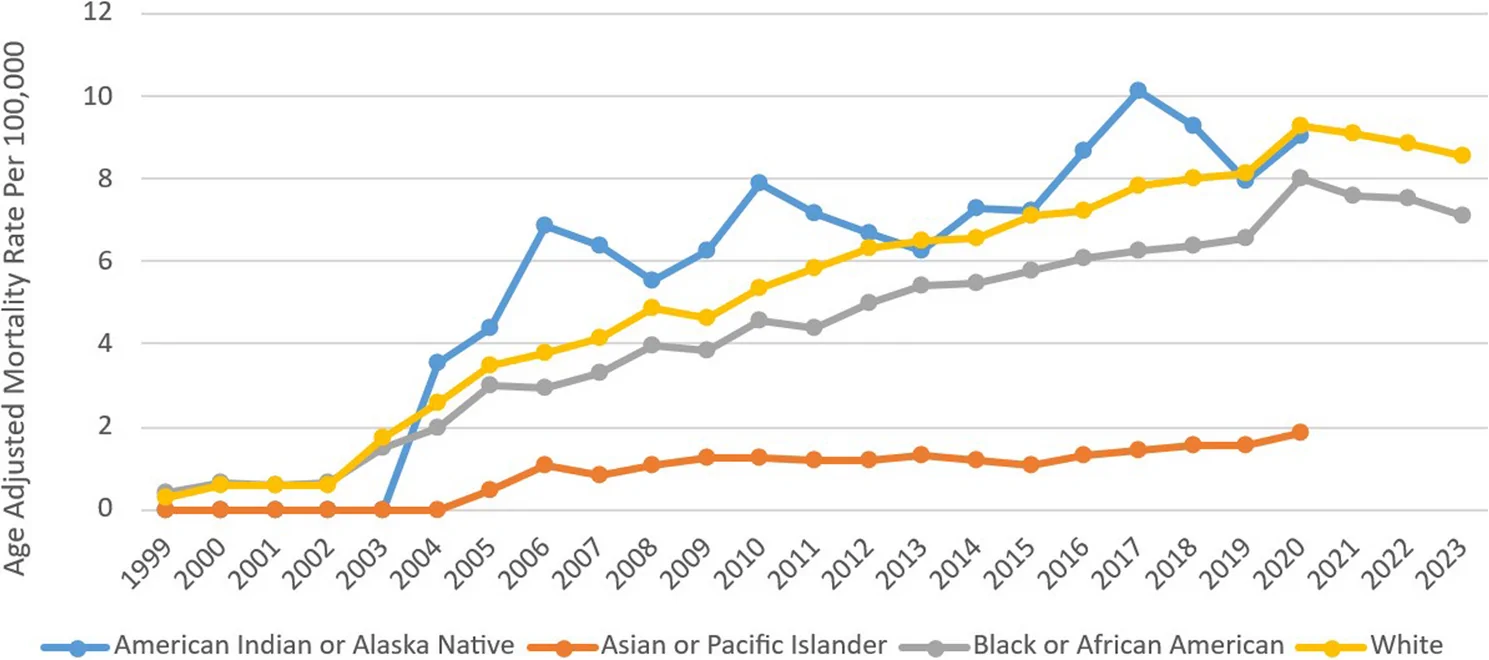
Psychoactive drugs and dementia-related age-adjusted mortality rates (AAMRs) per 100,000 individuals stratified by race/ethnicity in the United States, 2000 to 2023

As shown in Fig. 3; Tables 1 and 3, and Supplementary Table 2, for NH White individuals, AAMRs increased from 0.6 per 100,000 in 2000 to 8.57 per 100,000 in 2023. A steep increase was observed from 2000 to 2005 (APC: 54.42%, 95% CI: 38.02 to 72.75; *p* < 0.001), followed by a sustained increase from 2005 to 2020 (APC: 5.91%, 95% CI: 4.94 to 6.89; *p* < 0.001). A downtrend was noted thereafter from 2020 to 2023 (APC: −2.19%, 95% CI: −9.29 to 5.47; *p* = 0.54). Overall, this group exhibited the highest long-term burden, with an AAPC of 13.77% (95% CI: 10.97 to 16.65; *p* < 0.001).

Among Hispanic or Latino individuals, AAMRs rose from 0.22 per 100,000 in 2000 to 3.33 per 100,000 in 2023. Mortality increased sharply from 2000 to 2005 (APC: 55.12%, 95% CI: 25.87 to 91.15; *p* < 0.001), followed by a continued increase from 2005 to 2023 (APC: 3.70%, 95% CI: 2.74 to 4.67; *p* < 0.001). The overall trend was increasing (AAPC: 13.19%, 95% CI: 8.42 to 18.17; *p* < 0.001).

Among NH Black individuals, AAMRs increased from 0.66 per 100,000 in 2000 to 7.08 per 100,000 in 2023. Mortality rose rapidly from 2000 to 2005 (APC: 46.34%, 95% CI: 31.03 to 63.45; *p* < 0.001), followed by a continued increase from 2005 to 2020 (APC: 6.22%, 95% CI: 5.20 to 7.25; *p* < 0.001). A modest decline occurred after 2020 till 2023(APC: −2.02%, 95% CI: −8.94 to 5.42; *p* = 0.56). The overall trend was increasing (AAPC: 12.69%,95% CI: 9.95 to 15.50; *p* < 0.001).

### Age-group trends

**Fig. 4:**
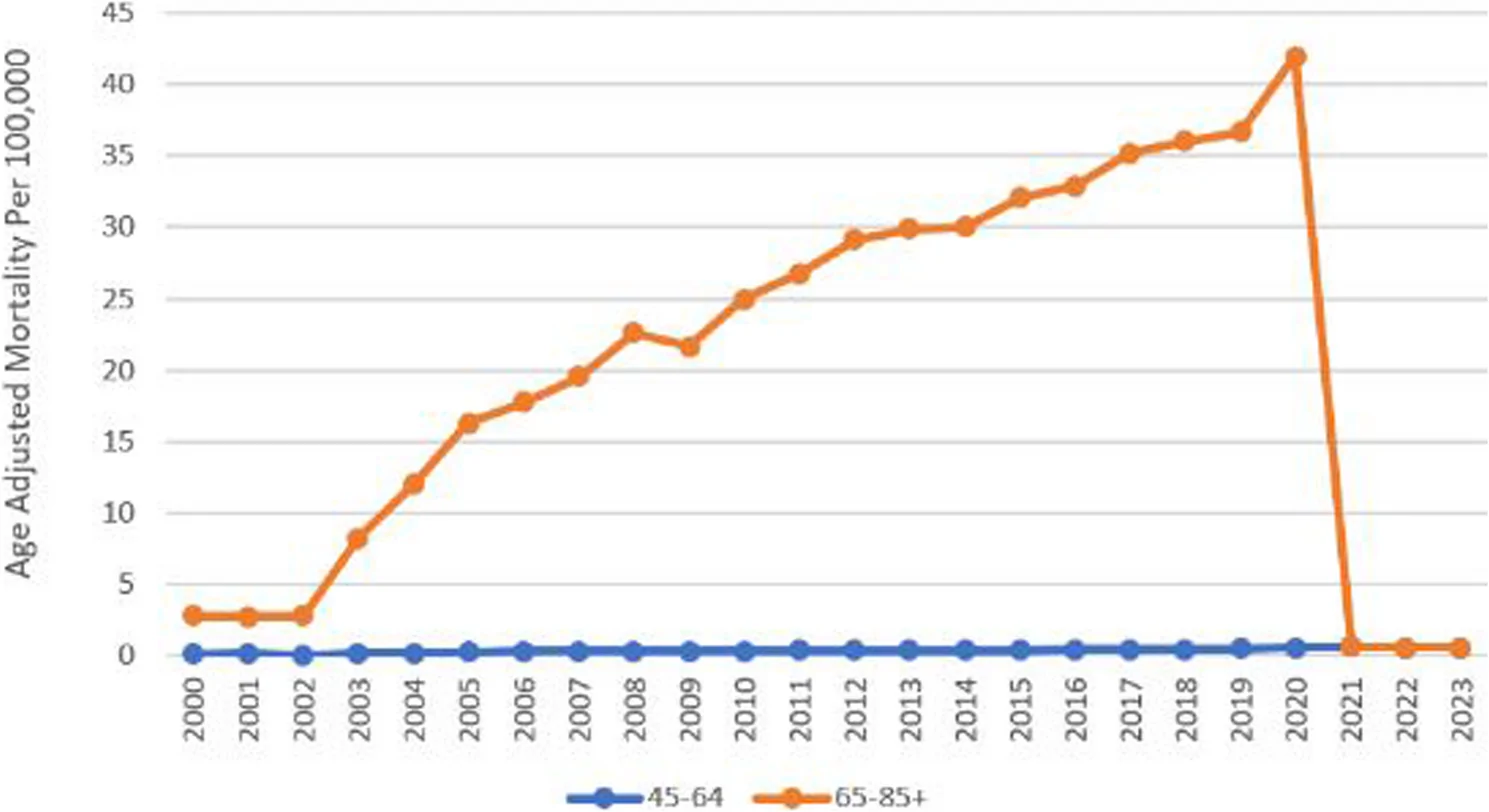
Psychoactive drugs and dementia-related age-adjusted mortality rates (AAMRs) per 100,000 individuals stratified by age in the United States, 2000 to 2023

Regarding age-group trends (Fig. 4; Table 3, and Supplementary Table 7), for individuals aged 45–64 years, age-adjusted mortality rates (AAMR) increased from 0.14 in 2000 to 0.61 in 2023 per 100,000; no joinpoints were identified, indicating a consistent linear increase across the entire study period. This group shows a stable upward trajectory over time with (AAPC = 5.54%; 95% CI: 4.74 to 6.35; *p* < 0.001). In contrast, the 65–85 + age group AAMRs increased from 2.79 in 2000 to 41.94 in 2020, followed by a sharp decline to 0.61 in 2023. Mortality rates surged dramatically during 2000–2005 (APC = 56.18%; 95% CI: 39.55 to 74.80; *p* < 0.001), followed by a continued increase from 2005 to 2020 (APC = 5.30%; 95% CI: 4.33 to 6.28; *p* < 0.001). However, a sharp decline was observed during 2020–2023 (APC = − 83.70%; 95% CI: −88.35 to − 77.21; *p* < 0.001). Consequently, the overall AAPC for this age group was declining (AAPC = − 10.06%; 95% CI: −14.16 to − 5.76; *p* < 0.001).

### Census region trends

**Fig. 5:**
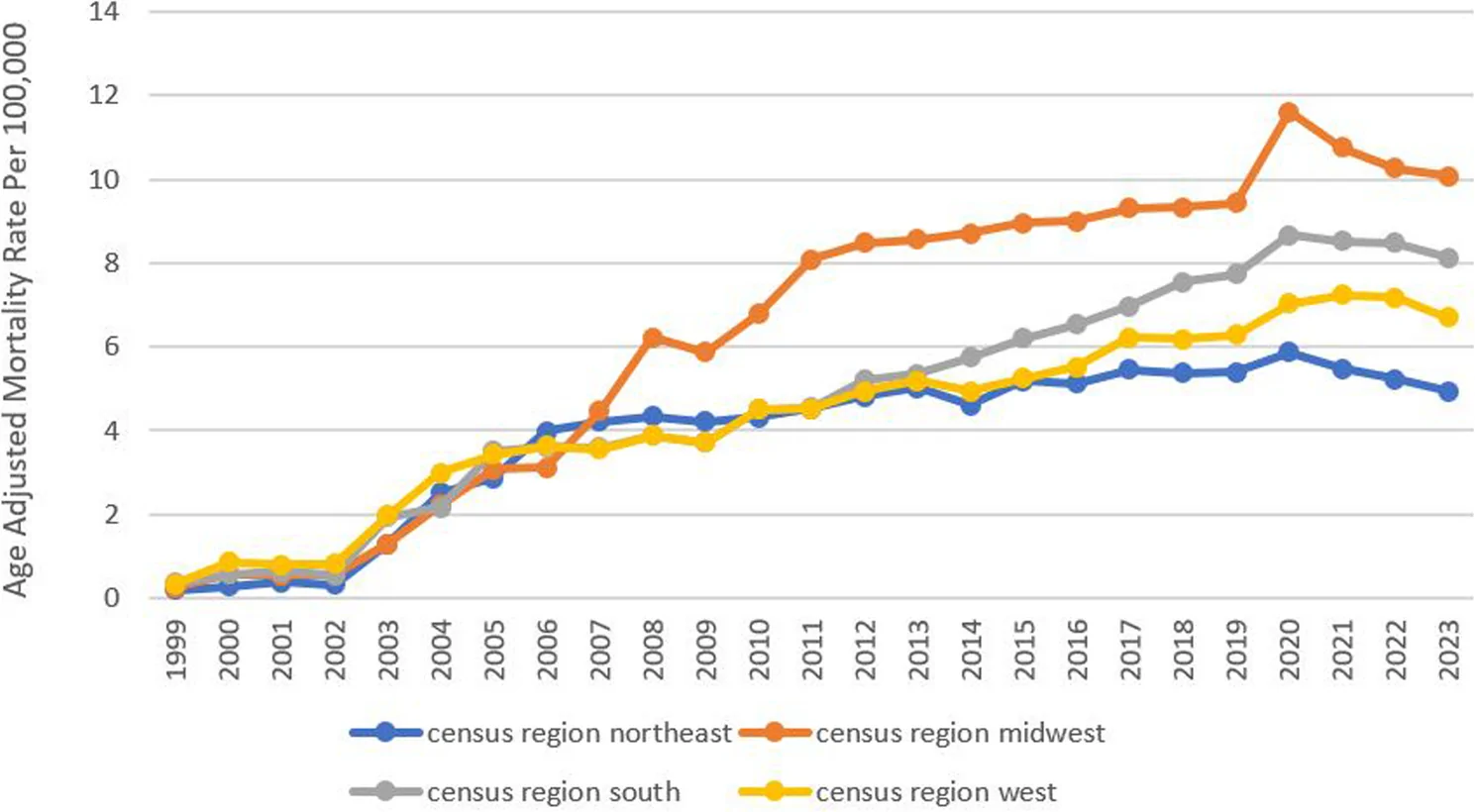
Psychoactive drugs and dementia-related age-adjusted mortality rates (AAMRs) per 100,000 individuals stratified by census region in the United States, 2000 to 2023

As summarized in Fig. 5; Table 3, and Supplementary Table 4, in the Northeast, Substance Use and Dementia-Related Mortality demonstrated a pronounced long-term increase between 2000 and 2023. The age-adjusted mortality rate (AAMR) increased from 0.29 per 100,000 population in 2000 to 4.95 per 100,000 population in 2023. A very steep early increase was observed from 2000 to 2004 (APC: 90.48%, 95% CI: 57.55 to 130.30; *p* < 0.001). This was followed by a non-significant transitional phase from 2004 to 2007 (APC: 18.92%, 95% CI: −4.85 to 48.63; *p* = 0.12). Subsequently, a sustained increase from 2007 to 2020 (APC: 2.37%, 95% CI: 1.27 to 3.49; *p* < 0.001). After 2020, a decline was noted through 2023 (APC: −4.42%, 95% CI: −12.20 to 4.06; *p* = 0.27). Despite recent attenuation, the overall trend remained strongly increasing, with an AAPC of 15.26% (95% CI: 10.57 to 20.15; *p* < 0.001).

The Midwest consistently exhibited the highest regional mortality across the United States. The AAMR increased from 0.58 per 100,000 in 2000 to 10.8 per 100,000 in 2023, representing the largest absolute increase among all census regions. A rapid early escalation was observed from 2000 to 2008 (APC: 38.15%, 95% CI: 27.90 to 49.23; *p* < 0.001). This was followed by a continued but minor increase from 2008 to 2023 (APC: 3.42%, 95% CI: 2.34 to 4.51; *p* < 0.001). Overall, the Midwest demonstrated a persistent increase, with an AAPC of 14.38% (95% CI: 11.45 to 17.38; *p* < 0.001).

In the South, PE-related mortality showed a substantial and sustained rise over the study period. The AAMR increased from 0.58 per 100,000 population in 2000 to 8.12 per 100,000 population in 2023. Joinpoint analysis revealed a sharp early increase from 2000 to 2005 (APC: 45.33%, 95% CI: 29.80 to 62.72; *p* < 0.001), followed by a prolonged period of continued growth from 2005 to 2020 (APC: 6.63%, 95% CI: 5.64 to 7.62; *p* < 0.001). From 2020 to 2023, the trend showed a decline (APC: −0.94%, 95% CI: −7.95 to 6.61; *p* = 0.79). Despite this recent stabilization, the overall trajectory remained upward, with an AAPC of 12.96% (95% CI: 10.17 to 15.83; *p* < 0.001).

The West also experienced a long-term increase in Substance Use and Dementia-Related Mortality. The AAMR rose from 0.86 per 100,000 in 2000 to 6.7 per 100,000 in 2023. An early rise was observed between 2000 and 2005 (APC: 37.94%, 95% CI: 24.22 to 53.16; *p* < 0.001). This was followed by a moderate increase from 2005 to 2023 (APC: 4.30%, 95% CI: 3.59 to 5.02; *p* < 0.001). Consequently, the West demonstrated a consistent upward trend across the entire study period, reflected by an AAPC of 10.84% (95% CI: 8.44 to 13.29; *p* < 0.001).

### State trends

**Fig. 6:**
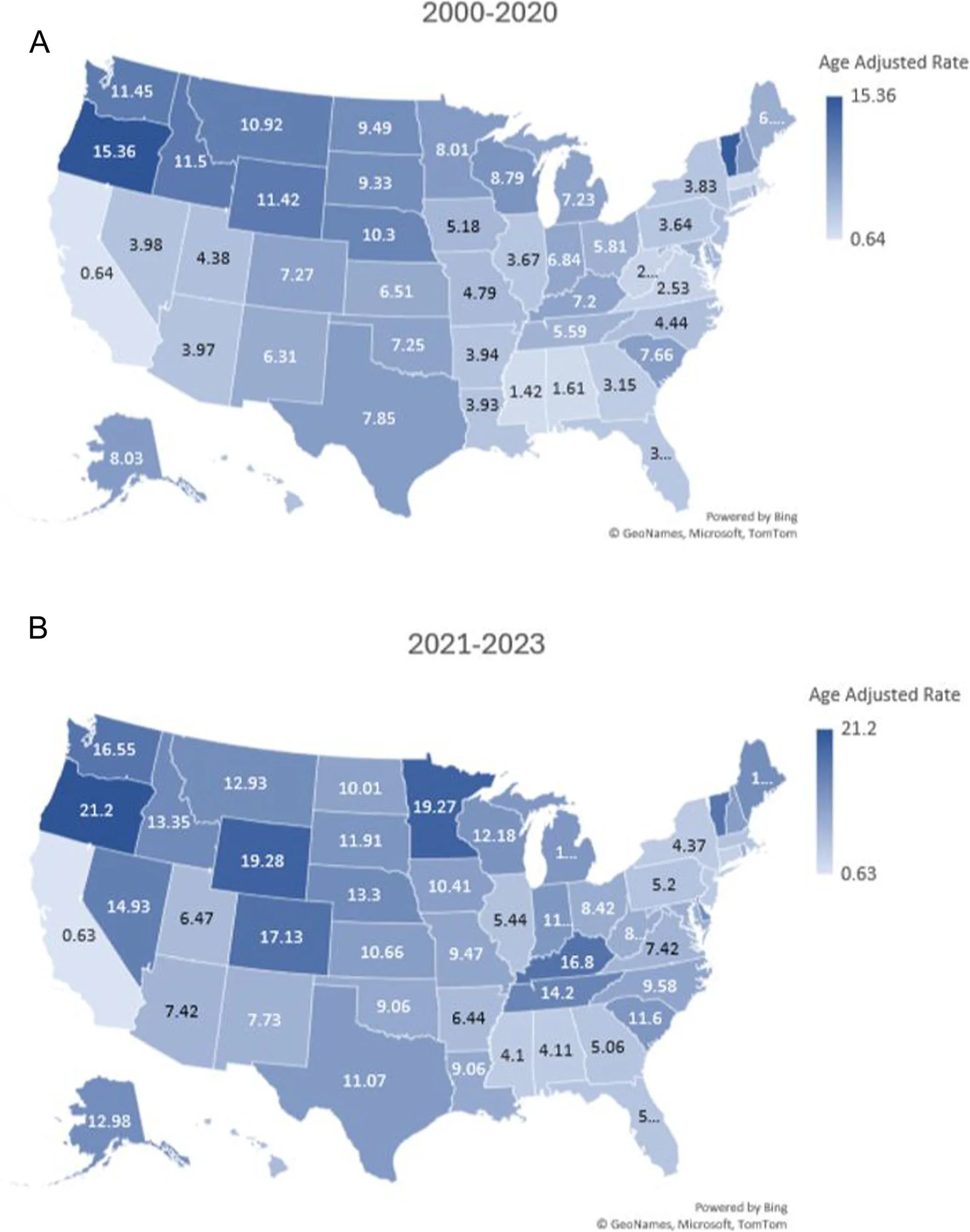
(**A**). Psychoactive drugs and dementia-related mortality distribution by states, 2000 to 2020. **B**. Psychoactive drugs and dementia-related mortality rates distribution by states, 2020 to 2023

**Fig. 7:**
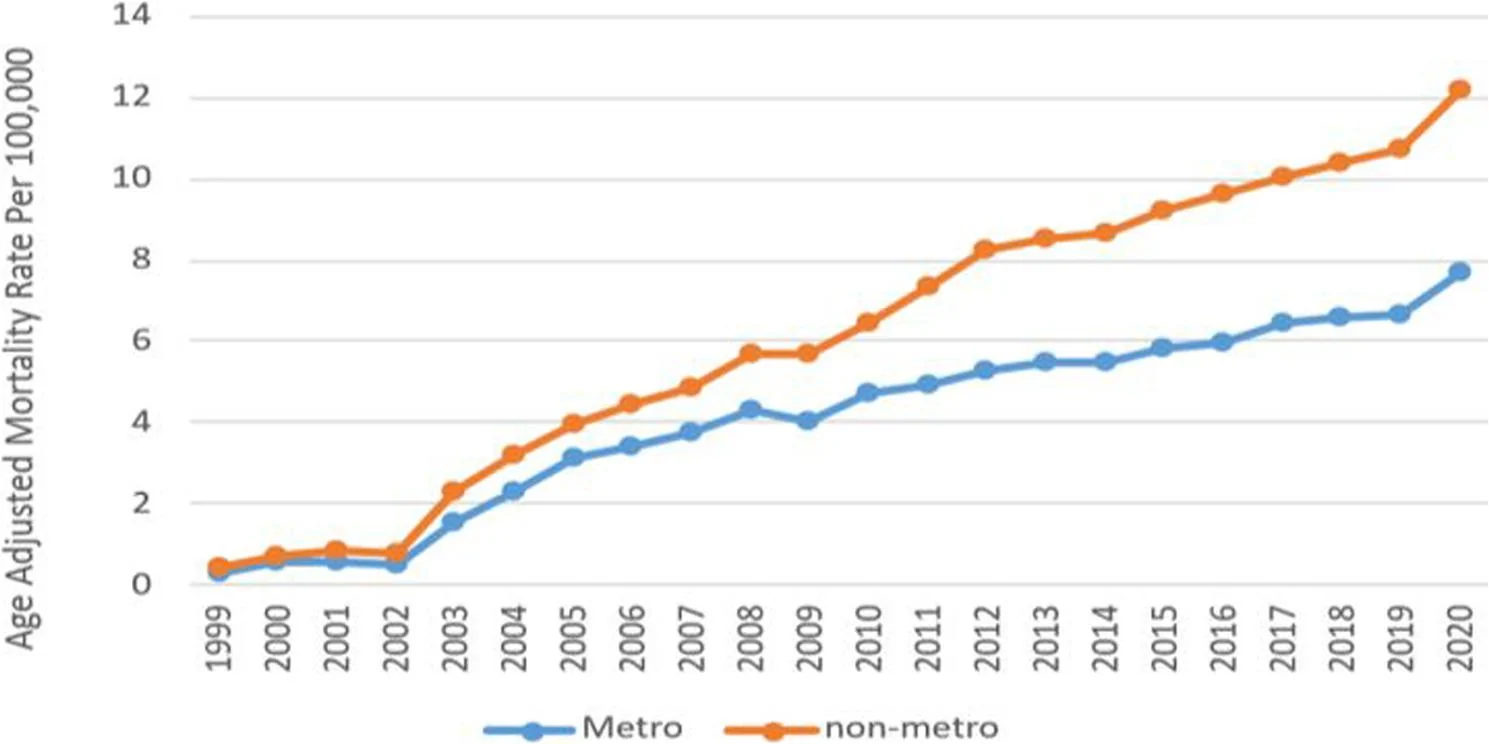
Psychoactive drugs and dementia-related age-adjusted mortality rates (AAMRs) per 100,000 individuals stratified by urbanization in the United States, 2000 to 2020 * data for urbanization AAMRs were unavailable for 2021 to 2023

As mapped in Fig. 6A and B, and 7, and Supplementary Table 3, throughout the study period, substantial statewide variation in age-adjusted Substance Use and Dementia-Related mortality rates was observed. From 2000 to 2020, states falling within the top 90th percentile for age-adjusted mortality rates included Oregon (15.36), Vermont (14.67), Idaho (11.50), Washington (11.45), Wyoming (11.42), and Montana (10.92), whereas states in the bottom 10th percentile included California (0.64), Mississippi (1.42), Alabama (1.61), Massachusetts (1.65), West Virginia (2.25), and Virginia (2.53).

In the subsequent period from 2021 to 2023, the geographic distribution of mortality shifted, with the highest age-adjusted mortality rates observed in Oregon (21.20), Wyoming (19.28), Minnesota (19.27), Colorado (17.13), Kentucky (16.80), and Washington (16.55). In contrast, California (0.63), the District of Columbia (3.22), Connecticut (3.52), Mississippi (4.10), Alabama (4.11), and New Jersey (4.32) ranked within the lowest 10th percentile of mortality rates during this period. Overall, interstate disparities persisted across both periods, with several states consistently ranking in either the highest or lowest mortality percentiles, indicating sustained geographic inequality in Substance Use and Dementia-Related Mortality burden.

### Urbanization trends

**Fig. 8:**
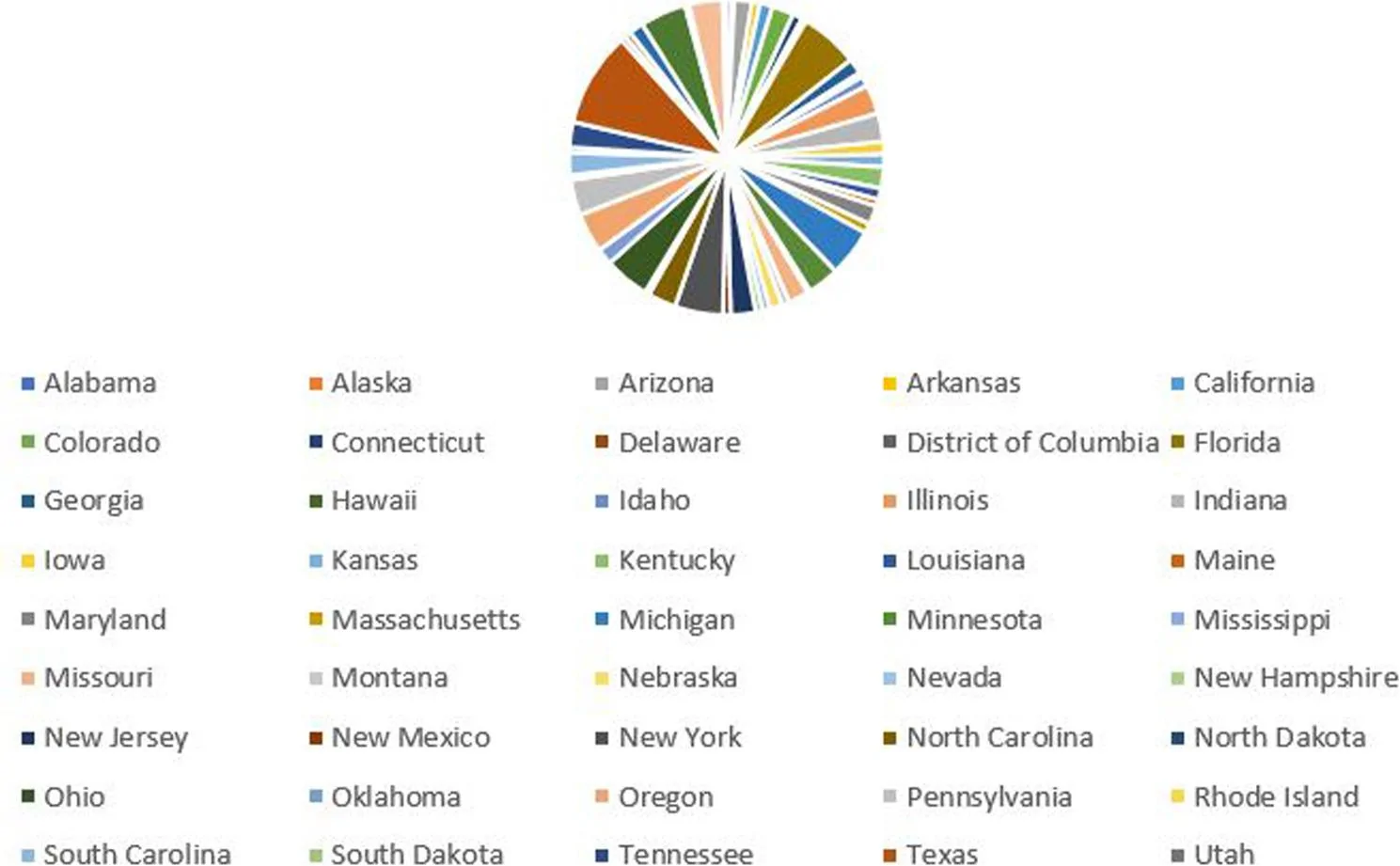
Psychoactive drugs and dementia-related age-adjusted mortality rates (AAMRs) per 100,000 individuals stratified by place of death in the United States, 2000 to 2023. Central Illustrations. Psychoactive drugs and dementia-related trends and disparities in mortality per 100,000 in the United States, 2000 to 2023. Summary of national patterns and demographic disparities with annual percent change (APC)

As illustrated in Fig. 8; Table 3, and Supplementary Table 5, in non-metropolitan areas, AAMRs increased from 0.71 per 100,000 in 2000 to 3.8 per 100,000 in 2023. Mortality surged from 2000 to 2004 (APC: 60.24%, 95% CI: 37.09 to 87.30; *p* < 0.001), followed by continued increases from 2004 to 2012 (APC: 11.10%, 95% CI: 7.88 to 14.40; *p* < 0.001) and 2012 to 2023 (APC: 4.65%, 95% CI: 2.93 to 6.40; *p* < 0.001). The overall trend was strongly increasing (AAPC: 16.72%, 95% CI: 13.17–20.37; *p* < 0.001).

In metropolitan areas, mortality increased from 0.56 per 100,000 to 3.5 per 100,000 over the study period. A sharp rise was observed from 2000 to 2005 (APC: 54.01%, 95% CI: 38.06 to 71.81; *p* < 0.001), followed by a continued increase thereafter (APC: 5.33%, 95% CI: 4.48 to 6.18; *p* < 0.001). The long-term trend remained upward (AAPC: 15.82%, 95% CI: 12.86–18.86; *p* < 0.001).

### Substance use and dementia-related mortality by place of death

As detailed in Supplementary Table 6, most deaths occurred in institutional settings. Nursing homes and long-term care facilities accounted for 42.96% of deaths, followed by deaths at home (26.18%) and inpatient medical facilities (15.96%). Hospice facilities represented 6.67%, outpatient or emergency departments 2.20%, and other locations 5.81%. Deaths classified as dead on arrival accounted for 0.14%, while 0.10% had unknown places of death.

## Discussion

Our nationwide multiple-cause-of-death analysis revealed a substantial increase in mortality rates over time, with AAMR increasing more than tenfold across the study period and a distinct rise during the early 2000s, followed by a slow but sustained increase through 2020.

These trends reflect the convergence of two clinically defined conditions. In DSM-5-TR terminology, major neurocognitive disorder refers to a significant decline in cognitive function that interferes with independence, while substance use disorder is characterized by a maladaptive pattern of substance use associated with clinically significant impairment. Although death certificate data do not capture formal diagnostic criteria, these constructs provide a useful clinical framework for interpreting the co-occurrence of dementia and substance-related mortality observed in this study [23].

The observed co-occurrence likely reflects both substance use as a risk factor for neurodegeneration and dementia as a condition that increases vulnerability to substance-related morbidity and mortality. In mortality data, substance-related causes may frequently represent the underlying cause of death, whereas dementia may act as a contributing condition, although the reverse relationship may also occur.

Several biological and clinical mechanisms may underlie the correlation between dementia and substance use. Substance use has long been associated with specific cognitive decline. Long-term alcohol consumption, including even moderate, chronic intake, has been linked to an increased risk of hippocampal atrophy, a critical structure in the formation of memories, indicating a direct negative effect on memory, with alcohol possibly acting as a precursor to dementia [24, 25]. Likewise, alcohol misuse is recognized as a modifiable risk factor for dementia, and current evidence suggests that up to approximately 40% of dementia cases may be attributable to modifiable risk factors overall [26]. Sedative-related complications, including falls, aspiration, and worsening cognition, have been associated with increased mortality among dementia patients. Particularly, benzodiazepines and antipsychotics, which are commonly prescribed to Alzheimer’s disease and related dementias (ADRD) for symptom management, carry significant risks in older adults, with antipsychotics having an FDA boxed warning for increased mortality in ADRD and benzodiazepines being linked to respiratory suppression, sedation, falls, and aspiration. Starting benzodiazepines is associated with a 41% higher risk of death at 180 days (HR 1.41), and starting antipsychotics is associated with a 16% higher risk (HR 1.16), with additional prescriptions of either medication further increasing mortality risk [27]. Benzodiazepines have been strongly associated with the development of delirium in older adults, particularly those with underlying cognitive impairment. Through potentiation of GABAergic activity and disruption of cholinergic pathways, these agents can precipitate acute confusional states characterized by inattention and altered consciousness. Delirium itself is an independent predictor of increased mortality and is associated with complications such as falls, aspiration, and functional decline [28, 29]. Further, substance use has been heavily linked to coexisting with other chronic conditions, including cardiovascular disease, metabolic disorders, chronic pulmonary disease, and cancer, which may collectively contribute to adverse health outcomes and increased mortality risk [30–32]. Together, these factors provide biologic and clinical plausibility for the increasing mortality burden observed among individuals with co-occurring dementia and substance use at the population level.

The overall annual trends are suggestive of a growing burden in recent years, with the observed trend reflective of the simultaneous overlap of two major public health concerns in the U.S., including population aging and a long-term increase in substance use-related mortality. During this period, the U.S. population aged ≥ 65 years nearly doubled from approximately 35 million in 2000 and is projected to reach about 71 million by 2030, while substance-related mortality has also risen substantially, with the age-adjusted rate of drug overdose deaths increasing from 8.9 deaths per 100,000 population in 2003 to 32.6 per 100,000 in 2022 [33, 34]. Further, prior literature has identified that a substantial proportion of the observed increases in dementia mortality can be attributed to changes in death certificate practices, which include a greater tendency to record dementia as a cause of death, as well as a decline in attribution to competing causes such as cardiovascular disease [35]. Our analysis revealed a distinct rise in the 2000s, which coincides with the initial phase of the prescription opioid epidemic, during which rapid expansion in opioid prescribing was associated with parallel increases in nonmedical use, treatment admissions, and overdose mortality [36]. This pharmaceutical-driven pattern provides a possible explanation for our trends, with this epidemic acting in conjunction with multiple other causes of these temporal trends. Similarly, the sustained increase observed throughout our study period likely reflects population ageing and declining early-life mortality, resulting in more individuals living longer with dementia; this expanding and longer-surviving population forms a larger, more vulnerable cohort at risk of adverse outcomes [26]. The non-significant decline observed from 2020 to 2023 likely reflects competing mortality during the COVID-19 pandemic. Deaths were more frequently attributed to COVID-19 rather than underlying chronic conditions. In addition, widespread disruptions in healthcare services for non-communicable diseases, including dementia care and addiction treatment, may have contributed to underdiagnosis, misclassification, and underreporting of these conditions on death certificates [37]. Understanding these temporal trends could therefore help in creating informed prevention and intervention strategies in terms of public health planning.

Sex-stratified analysis revealed higher mortality rates among males, although females presented with a greater relative increase over the study period. This could be explained by sociocultural expectations around substance use. Males have historically had a higher prevalence of substance use and substance use disorders (SUD), possibly contributing to a higher absolute mortality [38, 39]. However, recently, a narrowing in SUD prevalence between sexes has been observed [40], reflected in the rising mortality rates in women. Biological differences in substance metabolism may also contribute. Differences in alcohol metabolism and substance pharmacokinetics may result in higher systemic exposure and toxicity at lower intake levels in females, which could explain the recent rapid increase in females’ mortality rate [41]. This may be further compounded by longer life expectancy in females, increasing cumulative dementia risk over time [42]. This would indicate a greater lifetime risk of dementia [43], which may result in a greater proportion of older females contracting dementia and a relative increase in mortality observed over time. This also does not contradict the higher absolute mortality observed in males, which is likely to represent the higher prevalence of substance use and SUDs.

Race and ethnicity stratified analyses revealed overall increases in all groups over the study period. Notably, NH Black individuals had the highest long-term mortality burden, with NH White individuals showing the highest absolute mortality rates in later years. Hispanic or Latino individuals showed lower absolute rates, but a consistent increase was observed throughout. These discrepancies could be attributed to social determinants of health, particularly loneliness and lack of support systems, which disproportionately affect racial groups in the U.S., specifically NH Black and Hispanic or Latino individuals [44]. Loneliness and a fractured or absent social support system have been recognized as modifiable risk factors for dementia [45, 46] and may therefore represent an important pathway through which social conditions contribute to observed trends. Further, differential factors that could contribute to mortality rates in different races include access to and quality of healthcare, socioeconomic status, chronic comorbidity prevalence, and exposure/misuse of substances, each of which shows racial disparity in the U.S [44, 47–49].

Age-stratified analysis demonstrated mortality trends across midlife and older age groups. Among individuals in the 45–64-year age group, mortality rates increased linearly throughout the study period. This likely reflects increasing vulnerability to substance-related harm in midlife, rather than increasing substance use prevalence alone, with long-term effects of substance use (specifically alcohol) manifesting as cognitive impairment and reduced cognitive reserve, which may increase susceptibility to adverse outcomes when dementia is recorded as a contributing cause of death [50]. Additionally, over our study period, chronic comorbidity rates in this age group have increased [51–53], possibly leading to a greater number of deaths captured under multiple causes of death, including dementia and substance use. In contrast, individuals aged ≥ 65 years exhibited a pronounced early surge in mortality during the early 2000s, followed by sustained increases through 2020. This may be attributed to rapid population ageing during the study period, with the U.S. population aged ≥ 65 years increasing substantially over recent decades [54]. Further, the prevalence of dementia being recorded as a cause of death on death certificates has risen significantly over time [55], providing a possible explanation for the steady rise in mortality rates in this population. The sharp decline observed after 2020 in the older age group is likely reflective of the COVID-19 pandemic, including competing causes of mortality and cause-of-death displacement [56], as well as disruptions in healthcare access for dementia patients [57], rather than a true reduction in underlying disease burden.

Region and urbanization-stratified analyses demonstrated geographic heterogeneity in mortality trends. The Midwest consistently exhibited the highest mortality burden, whereas the Northeast demonstrated comparatively lower rates despite long-term increases. All census regions experienced upward trends, though the magnitude varied. Urbanization patterns similarly revealed increasing mortality in both metropolitan and non-metropolitan areas, with particularly strong growth in non-metropolitan regions. These trends could be attributed to variations across regions and levels of urbanization of substance use patterns, socioeconomic disparities, prevalence of chronic disease conditions, and healthcare and treatment access [58–61].

Most deaths in nursing homes and long-term care facilities likely reflect advanced dementia with a dependency on caretakers due to severe cognitive and functional impairment, potentially accelerated by coexisting substance use. Sedative medications, particularly benzodiazepines and antipsychotics, may further increase mortality risk through delirium, aspiration, and falls [28, 29]. Deaths at home may indicate variability in caregiving and palliative care access, with substance use contributing to delayed recognition of complications or acute events such as overdose [62, 63]. Inpatient deaths likely represent acute decompensations, while the low proportion of hospice deaths suggests underutilization of end-of-life care in this population [64].

These findings demonstrate a sustained increase in dementia and substance use–related mortality across demographic and geographic subgroups in the U.S., with observed heterogeneity reflecting the multifaceted nature of this growing burden. Contributing factors include population ageing, evolving substance use patterns, socioeconomic disparities, chronic disease prevalence, and healthcare access. As these factors act concurrently, solutions cannot rely on singular approaches; sustained improvement requires integrated public health strategies addressing both dementia and substance use.

This study is subject to limitations inherent to CDC WONDER data. These include potential misclassification of causes of death and underreporting of substance use due to reliance on death certificates. The absence of detailed clinical and socioeconomic variables also limits risk adjustment and introduces residual confounding. The observational nature of the dataset precludes causal inference and restricts interpretation to associations. Additionally, temporal trends may be influenced by changes in death certification and coding practices over time.

## Supplementary Information

Supplementary Material 1.

Supplementary Material 2.

## Data Availability

The datasets generated and/or analyzed during the current study are available from the corresponding author on reasonable request.
