# Supplementary material for: Rising trends in dementia and psychoactive drug-related mortality in the United States, 2000–2023. A retrospective analysis using CDC WONDER
Source: BMC Neurol. 2026 Jun 29;26:580. doi: 10.1186/s12883-026-05117-8 (PMC13573364; doi:10.1186/s12883-026-05117-8)
Supplement: Supplementary file 1 [file 12883_2026_5117_MOESM2_ESM.docx]

|  | **Age-Adjusted Rate (95% CI)** | | |
| --- | --- | --- | --- |
| **Year** | **Male** | **Female** | **Overall** |
| **2000** | 0.87 (0.80 - 0.94) | 0.41 (0.37 - 0.45) | 0.59 (0.56 - 0.63) |
| **2001** | 0.86 (0.79 - 0.93) | 0.42 (0.38 - 0.46) | 0.60 (0.56 - 0.63) |
| **2002** | 0.86 (0.79 - 0.93) | 0.41 (0.37 - 0.44) | 0.56 (0.53 - 0.59) |
| **2003** | 2.46 (2.34 - 2.58) | 1.20 (1.13 - 1.26) | 1.67 (1.61 - 1.73) |
| **2004** | 3.50 (3.36 - 3.64) | 1.77 (1.69 - 1.84) | 2.42 (2.35 - 2.49) |
| **2005** | 4.71 (4.55 - 4.87) | 2.44 (2.35 - 2.53) | 3.27 (3.19 - 3.35) |
| **2006** | 5.12 (4.95 - 5.28) | 2.66 (2.57 - 2.75) | 3.57 (3.49 - 3.66) |
| **2007** | 5.75 (5.57 - 5.92) | 2.81 (2.71 - 2.90) | 3.93 (3.84 - 4.01) |
| **2008** | 6.44 (6.26 - 6.62) | 3.37 (3.27 - 3.47) | 4.53 (4.44 - 4.62) |
| **2009** | 5.97 (5.80 - 6.14) | 3.30 (3.20 - 3.40) | 4.33 (4.24 - 4.42) |
| **2010** | 7.16 (6.97 - 7.34) | 3.65 (3.54 - 3.76) | 5.00 (4.91 - 5.10) |
| **2011** | 7.39 (7.21 - 7.58) | 4.09 (3.98 - 4.20) | 5.36 (5.26 - 5.46) |
| **2012** | 8.00 (7.81 - 8.19) | 4.46 (4.35 - 4.58) | 5.82 (5.72 - 5.92) |
| **2013** | 8.17 (7.98 - 8.36) | 4.59 (4.48 - 4.71) | 5.98 (5.88 - 6.08) |
| **2014** | 8.14 (7.96 - 8.33) | 4.65 (4.53 - 4.76) | 6.01 (5.91 - 6.11) |
| **2015** | 8.73 (8.54 - 8.92) | 4.90 (4.78 - 5.02) | 6.41 (6.31 - 6.51) |
| **2016** | 8.90 (8.71 - 9.09) | 5.04 (4.93 - 5.16) | 6.59 (6.49 - 6.70) |
| **2017** | 9.45 (9.26 - 9.64) | 5.42 (5.30 - 5.54) | 7.05 (6.94 - 7.15) |
| **2018** | 9.60 (9.41 - 9.79) | 5.61 (5.48 - 5.73) | 7.21 (7.11 - 7.32) |
| **2019** | 9.80 (9.61 - 9.99) | 5.69 (5.57 - 5.81) | 7.35 (7.25 - 7.46) |
| **2020** | 11.15 (10.95 - 11.35) | 6.50 (6.38 - 6.63) | 8.41 (8.30 - 8.52) |
| **2021** | 10.66 (10.46 - 10.86) | 6.38 (6.25 - 6.51) | 8.13 (8.01 - 8.24) |
| **2022** | 10.39 (10.20 - 10.58) | 6.31 (6.18 - 6.43) | 7.94 (7.83 - 8.05) |
| **2023** | 9.61 (9.42 - 9.79) | 6.23 (6.10 - 6.35) | 7.63 (7.52 - 7.73) |

**Supplemental Table 1:** Overall and Sex‐Stratified Substance Use and Dementia-Related Age-Adjusted Mortality Rates per 100,000 in the United States, 2000 to 2023.

|  | Age-Adjusted Rate (95% CI) | | |
| --- | --- | --- | --- |
| Year | **NH White** | **NH Black or African American** | **Hispanic or Latino** |
| 2000 | 0.60 (0.56 - 0.64) | 0.66 (0.53 - 0.81) | 0.22 (0.12 - 0.35) |
| 2001 | 0.62 (0.58 - 0.66) | 0.59 (0.47 - 0.73) | 0.35 (0.23 - 0.51) |
| 2002 | 0.59 (0.55 - 0.62) | 0.68 (0.54 - 0.81) | 0.29 (0.18 - 0.43) |
| 2003 | 1.75 (1.69 - 1.82) | 1.50 (1.30 - 1.70) | 1.14 (0.92 - 1.37) |
| 2004 | 2.57 (2.49 - 2.65) | 2.00 (1.77 - 2.24) | 1.25 (1.01 - 1.48) |
| 2005 | 3.47 (3.38 - 3.56) | 2.98 (2.69 - 3.26) | 1.80 (1.52 - 2.07) |
| 2006 | 3.78 (3.69 - 3.88) | 2.94 (2.67 - 3.22) | 2.25 (1.95 - 2.55) |
| 2007 | 4.18 (4.08 - 4.27) | 3.33 (3.04 - 3.62) | 2.23 (1.95 - 2.52) |
| 2008 | 4.85 (4.75 - 4.96) | 3.99 (3.68 - 4.31) | 2.11 (1.84 - 2.37) |
| 2009 | 4.64 (4.54 - 4.74) | 3.83 (3.52 - 4.13) | 2.15 (1.88 - 2.41) |
| 2010 | 5.36 (5.25 - 5.47) | 4.57 (4.24 - 4.90) | 2.41 (2.14 - 2.69) |
| 2011 | 5.83 (5.71 - 5.94) | 4.37 (4.05 - 4.68) | 2.38 (2.11 - 2.64) |
| 2012 | 6.35 (6.23 - 6.47) | 4.98 (4.65 - 5.31) | 2.74 (2.46 - 3.01) |
| 2013 | 6.52 (6.40 - 6.64) | 5.42 (5.08 - 5.76) | 2.77 (2.50 - 3.04) |
| 2014 | 6.56 (6.44 - 6.67) | 5.48 (5.15 - 5.82) | 3.01 (2.74 - 3.27) |
| 2015 | 7.08 (6.96 - 7.20) | 5.78 (5.44 - 6.12) | 2.76 (2.51 - 3.01) |
| 2016 | 7.25 (7.13 - 7.37) | 6.10 (5.76 - 6.44) | 3.01 (2.75 - 3.26) |
| 2017 | 7.80 (7.67 - 7.92) | 6.29 (5.95 - 6.63) | 3.10 (2.85 - 3.35) |
| 2018 | 8.00 (7.87 - 8.12) | 6.37 (6.04 - 6.71) | 3.46 (3.21 - 3.72) |
| 2019 | 8.16 (8.04 - 8.29) | 6.59 (6.25 - 6.92) | 3.47 (3.22 - 3.73) |
| 2020 | 9.30 (9.17 - 9.43) | 8.03 (7.66 - 8.40) | 4.17 (3.90 - 4.44) |
| 2021 | 9.11 (8.98 - 9.25) | 7.60 (7.24 - 7.96) | 3.56 (3.31 - 3.82) |
| 2022 | 8.88 (8.75 - 9.01) | 7.52 (7.16 - 7.87) | 3.75 (3.50 - 4.00) |
| 2023 | 8.57 (8.44 - 8.70) | 7.08 (6.74 - 7.42) | 3.33 (3.10 - 3.57) |

NH = non-Hispanic.

**Supplemental Table 2:** Substance Use and Dementia-Related Mortality Rates per 100,000 Stratified by Race in the United States, 2000 to 2023.

| **State** | **Age-Adjusted Rate (95%CI)** | |
| --- | --- | --- |
|  | **2000-2020** | **2021-2023** |
| **Alabama** | 1.61 (1.52 - 1.71) | 4.11 (3.75 - 4.47) |
| **Alaska** | 8.03 (7.22 - 8.83) | 12.98 (10.82 - 15.14) |
| **Arizona** | 3.97 (3.84 - 4.10) | 7.42 (7.03 - 7.81) |
| **Arkansas** | 3.94 (3.76 - 4.12) | 6.44 (5.86 - 7.02) |
| **California** | 0.64 (0.62 - 0.66) | 0.63 (0.58 - 0.69) |
| **Colorado** | 7.27 (7.05 - 7.49) | 17.13 (16.37 - 17.90) |
| **Connecticut** | 3.95 (3.80 - 4.11) | 3.52 (3.14 - 3.90) |
| **Delaware** | 5.25 (4.87 - 5.64) | 13.13 (11.75 - 14.52) |
| **District of Columbia** | 2.67 (2.31 - 3.03) | 3.22 (2.32 - 4.35) |
| **Florida** | 3.57 (3.51 - 3.63) | 5.13 (4.96 - 5.30) |
| **Georgia** | 3.15 (3.04 - 3.26) | 5.06 (4.75 - 5.36) |
| **Hawaii** | 4.32 (4.05 - 4.58) | 6.53 (5.75 - 7.31) |
| **Idaho** | 11.50 (11.04 - 11.96) | 13.35 (12.24 - 14.47) |
| **Illinois** | 3.67 (3.58 - 3.76) | 5.44 (5.17 - 5.70) |
| **Indiana** | 6.84 (6.67 - 7.01) | 11.52 (10.98 - 12.06) |
| **Iowa** | 5.18 (4.98 - 5.37) | 10.41 (9.71 - 11.11) |
| **Kansas** | 6.51 (6.27 - 6.74) | 10.66 (9.89 - 11.43) |
| **Kentucky** | 7.20 (6.99 - 7.42) | 16.80 (16.00 - 17.60) |
| **Louisiana** | 3.93 (3.78 - 4.09) | 9.06 (8.47 - 9.65) |
| **Maine** | 6.18 (5.86 - 6.50) | 12.83 (11.73 - 13.93) |
| **Maryland** | 5.38 (5.22 - 5.54) | 8.34 (7.86 - 8.81) |
| **Massachusetts** | 1.65 (1.57 - 1.73) | 5.30 (4.96 - 5.65) |
| **Michigan** | 7.23 (7.09 - 7.36) | 11.63 (11.20 - 12.05) |
| **Minnesota** | 8.01 (7.82 - 8.21) | 19.27 (18.53 - 20.00) |
| **Mississippi** | 1.42 (1.30 - 1.54) | 4.10 (3.61 - 4.58) |
| **Missouri** | 4.79 (4.65 - 4.93) | 9.47 (8.98 - 9.96) |
| **Montana** | 10.92 (10.41 - 11.43) | 12.93 (11.60 - 14.26) |
| **Nebraska** | 10.30 (9.93 - 10.67) | 13.30 (12.24 - 14.36) |
| **Nevada** | 3.98 (3.76 - 4.21) | 14.93 (13.98 - 15.88) |
| **New Hampshire** | 8.13 (7.73 - 8.52) | 10.65 (9.60 - 11.71) |
| **New Jersey** | 4.46 (4.35 - 4.57) | 4.32 (4.05 - 4.59) |
| **New Mexico** | 6.31 (6.02 - 6.60) | 7.73 (6.98 - 8.48) |
| **New York** | 3.83 (3.76 - 3.89) | 4.37 (4.19 - 4.56) |
| **North Carolina** | 4.44 (4.32 - 4.55) | 9.58 (9.19 - 9.97) |
| **North Dakota** | 9.49 (8.93 - 10.04) | 10.01 (8.57 - 11.46) |
| **Ohio** | 5.81 (5.70 - 5.92) | 8.42 (8.08 - 8.75) |
| **Oklahoma** | 7.25 (7.03 - 7.48) | 9.06 (8.43 - 9.69) |
| **Oregon** | 15.36 (15.05 - 15.68) | 21.20 (20.31 - 22.09) |
| **Pennsylvania** | 3.64 (3.56 - 3.72) | 5.20 (4.96 - 5.43) |
| **Rhode Island** | 5.74 (5.40 - 6.09) | 7.97 (6.94 - 8.99) |
| **South Carolina** | 7.66 (7.45 - 7.88) | 11.60 (11.00 - 12.19) |
| **South Dakota** | 9.33 (8.82 - 9.84) | 11.91 (10.46 - 13.36) |
| **Tennessee** | 5.59 (5.44 - 5.75) | 14.20 (13.61 - 14.79) |
| **Texas** | 7.85 (7.75 - 7.96) | 11.07 (10.78 - 11.36) |
| **Utah** | 4.38 (4.13 - 4.63) | 6.47 (5.77 - 7.17) |
| **Vermont** | 14.67 (13.92 - 15.41) | 16.36 (14.47 - 18.24) |
| **Virginia** | 2.53 (2.44 - 2.63) | 7.42 (7.04 - 7.80) |
| **Washington** | 11.45 (11.23 - 11.67) | 16.55 (15.94 - 17.17) |
| **West Virginia** | 2.25 (2.09 - 2.42) | 8.42 (7.61 - 9.23) |
| **Wisconsin** | 8.79 (8.59 - 8.98) | 12.18 (11.62 - 12.75) |
| **Wyoming** | 11.42 (10.65 - 12.19) | 19.28 (16.91 - 21.65) |

**Supplemental Table 3:** Substance Use and Dementia-Related Age-Adjusted Mortality Rates per 100,000 Stratified by States in the United States, 2000 to 2023.

| **Census Region** | **Year** | **Age-Adjusted Rate (95% CI)** |
| --- | --- | --- |
| **Northeast** |  |  |
| **Northeast** | 2000 | 0.29 (0.23 - 0.34) |
| **Northeast** | 2001 | 0.38 (0.32 - 0.44) |
| **Northeast** | 2002 | 0.33 (0.28 - 0.39) |
| **Northeast** | 2003 | 1.30 (1.19 - 1.41) |
| **Northeast** | 2004 | 2.52 (2.37 - 2.68) |
| **Northeast** | 2005 | 2.88 (2.72 - 3.05) |
| **Northeast** | 2006 | 3.99 (3.80 - 4.18) |
| **Northeast** | 2007 | 4.22 (4.03 - 4.42) |
| **Northeast** | 2008 | 4.34 (4.14 - 4.54) |
| **Northeast** | 2009 | 4.22 (4.03 - 4.42) |
| **Northeast** | 2010 | 4.33 (4.14 - 4.53) |
| **Northeast** | 2011 | 4.52 (4.32 - 4.72) |
| **Northeast** | 2012 | 4.82 (4.61 - 5.02) |
| **Northeast** | 2013 | 5.02 (4.81 - 5.23) |
| **Northeast** | 2014 | 4.61 (4.41 - 4.81) |
| **Northeast** | 2015 | 5.20 (4.99 - 5.41) |
| **Northeast** | 2016 | 5.13 (4.92 - 5.34) |
| **Northeast** | 2017 | 5.46 (5.25 - 5.67) |
| **Northeast** | 2018 | 5.38 (5.17 - 5.59) |
| **Northeast** | 2019 | 5.40 (5.19 - 5.61) |
| **Northeast** | 2020 | 5.87 (5.65 - 6.08) |
| **Northeast** | 2021 | 5.47 (5.26 - 5.68) |
| **Northeast** | 2022 | 5.24 (5.04 - 5.44) |
| **Northeast** | 2023 | 4.95 (4.76 - 5.15) |
| **Midwest** |  |  |
| **Midwest** | 2000 | 0.58 (0.50 - 0.65) |
| **Midwest** | 2001 | 0.56 (0.49 - 0.63) |
| **Midwest** | 2002 | 0.58 (0.51 - 0.65) |
| **Midwest** | 2003 | 1.30 (1.20 - 1.41) |
| **Midwest** | 2004 | 2.23 (2.09 - 2.36) |
| **Midwest** | 2005 | 3.08 (2.92 - 3.24) |
| **Midwest** | 2006 | 3.11 (2.95 - 3.27) |
| **Midwest** | 2007 | 4.47 (4.28 - 4.66) |
| **Midwest** | 2008 | 6.23 (6.00 - 6.45) |
| **Midwest** | 2009 | 5.88 (5.66 - 6.10) |
| **Midwest** | 2010 | 6.78 (6.54 - 7.01) |
| **Midwest** | 2011 | 8.08 (7.83 - 8.33) |
| **Midwest** | 2012 | 8.49 (8.23 - 8.75) |
| **Midwest** | 2013 | 8.57 (8.31 - 8.82) |
| **Midwest** | 2014 | 8.70 (8.44 - 8.96) |
| **Midwest** | 2015 | 8.95 (8.69 - 9.21) |
| **Midwest** | 2016 | 9.00 (8.74 - 9.26) |
| **Midwest** | 2017 | 9.32 (9.06 - 9.58) |
| **Midwest** | 2018 | 9.33 (9.08 - 9.59) |
| **Midwest** | 2019 | 9.43 (9.18 - 9.69) |
| **Midwest** | 2020 | 11.61 (11.33 - 11.89) |
| **Midwest** | 2021 | 10.75 (10.47 - 11.03) |
| **Midwest** | 2022 | 10.27 (10.01 - 10.54) |
| **Midwest** | 2023 | 10.08 (9.82 - 10.34) |
| **South** |  |  |
| **South** | 2000 | 0.58 (0.52 - 0.64) |
| **South** | 2001 | 0.63 (0.57 - 0.69) |
| **South** | 2002 | 0.56 (0.50 - 0.62) |
| **South** | 2003 | 1.93 (1.83 - 2.04) |
| **South** | 2004 | 2.16 (2.04 - 2.27) |
| **South** | 2005 | 3.52 (3.37 - 3.66) |
| **South** | 2006 | 3.62 (3.47 - 3.76) |
| **South** | 2007 | 3.60 (3.45 - 3.74) |
| **South** | 2008 | 3.88 (3.74 - 4.03) |
| **South** | 2009 | 3.72 (3.58 - 3.86) |
| **South** | 2010 | 4.51 (4.36 - 4.67) |
| **South** | 2011 | 4.54 (4.39 - 4.69) |
| **South** | 2012 | 5.22 (5.06 - 5.38) |
| **South** | 2013 | 5.36 (5.20 - 5.52) |
| **South** | 2014 | 5.75 (5.58 - 5.91) |
| **South** | 2015 | 6.20 (6.04 - 6.37) |
| **South** | 2016 | 6.54 (6.37 - 6.71) |
| **South** | 2017 | 6.97 (6.80 - 7.15) |
| **South** | 2018 | 7.55 (7.37 - 7.72) |
| **South** | 2019 | 7.74 (7.56 - 7.91) |
| **South** | 2020 | 8.66 (8.48 - 8.84) |
| **South** | 2021 | 8.53 (8.34 - 8.72) |
| **South** | 2022 | 8.48 (8.30 - 8.66) |
| **South** | 2023 | 8.12 (7.94 - 8.29) |
| **West** |  |  |
| **West** | 2000 | 0.86 (0.77 - 0.96) |
| **West** | 2001 | 0.81 (0.72 - 0.91) |
| **West** | 2002 | 0.82 (0.73 - 0.92) |
| **West** | 2003 | 1.99 (1.84 - 2.13) |
| **West** | 2004 | 3.00 (2.83 - 3.17) |
| **West** | 2005 | 3.43 (3.25 - 3.62) |
| **West** | 2006 | 3.63 (3.44 - 3.81) |
| **West** | 2007 | 3.55 (3.37 - 3.74) |
| **West** | 2008 | 3.89 (3.70 - 4.08) |
| **West** | 2009 | 3.73 (3.55 - 3.92) |
| **West** | 2010 | 4.50 (4.30 - 4.70) |
| **West** | 2011 | 4.52 (4.32 - 4.71) |
| **West** | 2012 | 4.94 (4.73 - 5.14) |
| **West** | 2013 | 5.18 (4.97 - 5.38) |
| **West** | 2014 | 4.94 (4.74 - 5.13) |
| **West** | 2015 | 5.25 (5.05 - 5.44) |
| **West** | 2016 | 5.52 (5.32 - 5.73) |
| **West** | 2017 | 6.23 (6.02 - 6.44) |
| **West** | 2018 | 6.18 (5.97 - 6.38) |
| **West** | 2019 | 6.29 (6.08 - 6.49) |
| **West** | 2020 | 7.03 (6.82 - 7.25) |
| **West** | 2021 | 7.23 (7.00 - 7.46) |
| **West** | 2022 | 7.18 (6.96 - 7.40) |
| **West** | 2023 | 6.70 (6.49 - 6.91) |

**Supplemental Table 4:** Substance Use and Dementia-Related Mortality Rates per 100,000, Stratified by Census Region in the United States, 2000 to 2023.

|  | **Age-Adjusted Rate (95% CI)** | |
| --- | --- | --- |
| **Year** | **Urban** | **Rural** |
| **2000** | 0.56 (0.52 - 0.60) | 0.71 (0.62 - 0.80) |
| **2001** | 0.54 (0.50 - 0.57) | 0.82 (0.73 - 0.92) |
| **2002** | 0.52 (0.48 - 0.55) | 0.75 (0.66 - 0.84) |
| **2003** | 1.51 (1.45 - 1.58) | 2.31 (2.15 - 2.47) |
| **2004** | 2.26 (2.18 - 2.33) | 3.16 (2.98 - 3.35) |
| **2005** | 3.10 (3.02 - 3.19) | 3.98 (3.77 - 4.19) |
| **2006** | 3.40 (3.31 - 3.49) | 4.41 (4.20 - 4.63) |
| **2007** | 3.72 (3.63 - 3.81) | 4.86 (4.63 - 5.08) |
| **2008** | 4.28 (4.18 - 4.38) | 5.68 (5.44 - 5.92) |
| **2009** | 4.04 (3.95 - 4.14) | 5.67 (5.42 - 5.91) |
| **2010** | 4.68 (4.57 - 4.78) | 6.47 (6.21 - 6.72) |
| **2011** | 4.92 (4.81 - 5.02) | 7.32 (7.05 - 7.59) |
| **2012** | 5.30 (5.20 - 5.41) | 8.27 (7.98 - 8.55) |
| **2013** | 5.44 (5.34 - 5.55) | 8.50 (8.21 - 8.79) |
| **2014** | 5.46 (5.36 - 5.57) | 8.65 (8.37 - 8.94) |
| **2015** | 5.84 (5.73 - 5.95) | 9.21 (8.91 - 9.50) |
| **2016** | 5.97 (5.86 - 6.08) | 9.61 (9.31 - 9.91) |
| **2017** | 6.43 (6.32 - 6.54) | 10.02 (9.72 - 10.32) |
| **2018** | 6.57 (6.46 - 6.68) | 10.42 (10.12 - 10.73) |
| **2019** | 6.65 (6.54 - 6.76) | 10.75 (10.44 - 11.06) |
| **2020** | 7.66 (7.54 - 7.77) | 12.20 (11.87 - 12.52) |

**Supplemental Table 5:** Substance Use and Dementia-Related Age-Adjusted Mortality Rates per 100,000 in the United States stratified by Urban-Rural Classification, 2000-2020.

| **Place of Death** | **Deaths** | **Percentage** |
| --- | --- | --- |
| Medical Facility - Inpatient | 45191 | 15.96% |
| Medical Facility - Outpatient or ER | 6226 | 2.20% |
| Medical Facility - Dead on Arrival | 386 | 0.14% |
| Medical Facility - Status unknown | 0 | 0% |
| Decedent's home | 74125 | 26.18% |
| Hospice facility | 18876 | 6.67% |
| Nursing home/long term care | 121648 | 42.96% |
| Other | 16450 | 5.81% |
| Place of death unknown | 292 | 0.10% |

**Supplemental Table 6**: Number of Deaths by place of death due to Dementia and Psychoactive Drug in the United States, 2000-2023.

|  | **Crude Mortality Rates (95% CI)** | | |
| --- | --- | --- | --- |
| **Year** | **Age 25-44** | **Age 45-64** | **Age 65+** |
| 2000 | Suppressed | 0.11 (0.08 - 0.14) | 2.77 (2.59 - 2.94) |
| 2001 | Unreliable | 0.14 (0.11 - 0.17) | 2.76 (2.59 - 2.94) |
| 2002 | Unreliable | 0.07 (0.05 - 0.09) | 2.83 (2.65 - 3.00) |
| 2003 | Suppressed | 0.16 (0.13 - 0.19) | 8.28 (7.99 - 8.58) |
| 2004 | Suppressed | 0.20 (0.17 - 0.23) | 12.17 (11.81 - 12.53) |
| 2005 | Suppressed | 0.25 (0.22 - 0.29) | 16.59 (16.17 - 17.00) |
| 2006 | Suppressed | 0.31 (0.27 - 0.35) | 18.22 (17.78 - 18.65) |
| 2007 | Suppressed | 0.33 (0.29 - 0.37) | 20.02 (19.57 - 20.47) |
| 2008 | Unreliable | 0.37 (0.33 - 0.41) | 22.94 (22.46 - 23.42) |
| 2009 | Suppressed | 0.31 (0.27 - 0.34) | 21.91 (21.45 - 22.37) |
| 2010 | Suppressed | 0.36 (0.32 - 0.40) | 25.28 (24.78 - 25.77) |
| 2011 | Unreliable | 0.42 (0.37 - 0.46) | 27.05 (26.55 - 27.55) |
| 2012 | Suppressed | 0.46 (0.41 - 0.50) | 28.93 (28.42 - 29.44) |
| 2013 | Suppressed | 0.48 (0.43 - 0.52) | 29.34 (28.83 - 29.84) |
| 2014 | Suppressed | 0.49 (0.45 - 0.54) | 29.21 (28.72 - 29.70) |
| 2015 | Suppressed | 0.51 (0.46 - 0.56) | 30.92 (30.43 - 31.42) |
| 2016 | Suppressed | 0.54 (0.49 - 0.59) | 31.47 (30.97 - 31.96) |
| 2017 | Unreliable | 0.57 (0.52 - 0.63) | 33.36 (32.86 - 33.86) |
| 2018 | Suppressed | 0.62 (0.57 - 0.68) | 34.11 (33.61 - 34.61) |
| 2019 | Suppressed | 0.63 (0.58 - 0.68) | 34.39 (33.90 - 34.89) |
| 2020 | Suppressed | 0.78 (0.72 - 0.84) | 38.99 (38.47 - 39.50) |
| 2021 | Unreliable | 0.84 (0.77 - 0.90) | 35.68 (35.18 - 36.17) |
| 2022 | Unreliable | 0.81 (0.75 - 0.87) | 36.16 (35.67 - 36.65) |
| 2023 | Unreliable | 0.77 (0.70 - 0.83) | 33.90 (33.42 - 34.37) |

**Supplemental Table 7:** Substance Use and Dementia-Related Crude Mortality Rates per 1,00,000, Stratified by Age-Groups in Adults in the United States, 2020 to 2023.
